# Multi-dimensional spatial-temporal projection ultrafast compressed imaging

**DOI:** 10.1038/s41377-026-02317-2

**Published:** 2026-06-29

**Authors:** Yizhao Meng, Pengfei Zhang, Jiaxin Yin, Yu Lu, Kaiduan Yue, Fei Yin, Qing Yang, Feng Chen

**Affiliations:** 1 State Key Laboratory for Manufacturing System Engineering and Shaanxi Key Laboratory of Photonics Technology for Information, School of Electronic Science and Engineering, Xi’an Jiaotong University, Xi’an, 710049 China; 2 School of Instrument Science and technology, Xi’an Jiaotong University, Xi’an, 710049 China; 3 Xi’an Institute of Optics and Precision Mechanics (XIOPM), Chinese Academy of Sciences (CAS), Xi’an, 710119 China

**Keywords:** Imaging and sensing, Ultrafast photonics

## Abstract

Studying various non-repeatable transient phenomena such as the transfer of photosynthetic energy and protein folding is crucial for gaining in-depth insights into multiple disciplines, while single-shot ultrafast compressed imaging provides a potent method for revealing the ultrafast dynamic process. The principle of this technology involves projecting encoded transient phenomena along a one-dimensional spatial axis onto the focal plane array and reconstructing the transient data via compressed sensing, enabling an extremely high imaging sequence depth at THz frame rates. However, this imaging method relying on single-dimensional spatial-temporal projection (SSP) suffers from severe directional constraints on reconstruction resolution, due to the suppressing of spatial features along non-projection direction. Consequently, this bottleneck seriously restricts the observation of complex transient features, hindering this technology to balance both high sequence depth and ultra-high spatial resolution. In this paper, we propose a multi-dimensional spatial-temporal projection ultrafast compressed imaging technique (MSP), which integrates multi-angle spatial-temporal data projection module into traditional SSP system. By computationally coupling projection images from both systems, MSP effectively restores omnidirectional frequency information, significantly enhancing spatial resolution. Static experiments demonstrate that both the lateral resolution and longitudinal resolution of MSP have reached 813 lp mm^-1^ (620 nm spatial resolution). Using MSP, we have successfully observed the transient process of femtosecond laser-material interaction with sub-micron all-directional accuracy under a 60-frame acquisition in a single-shot. MSP provides a new method for observing complex and changeable transient processes, and shows important potential in promoting the basic research of transient phenomena.

## Abstract (web summary)

An ultrafast compressed imaging technique utilizing multi-dimensional spatial-temporal projection can achieve isotropic reconstruction, successfully observing complex transient phenomena with sub-micron resolution and ultra-high sequence depth.

## Graphical abstract

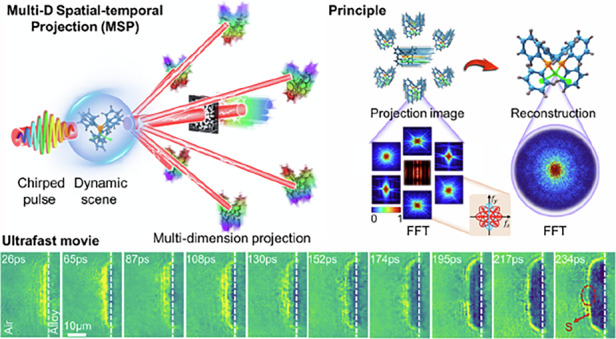

## Introduction

Since the advent of ultrafast imaging technology, there has been a progressively clearer understanding of transient changes in the microscopic world. The well-known phenomena such as transfer of photosynthetic energy^1,2^, protein structure formation^3,4^ and laser ablation^5,6^ occur at extremely fast time scales (fs-ns), where ultrafast imaging plays an important role. For instance, visualizing the dynamic process of femtosecond laser ablation material not only enhances our comprehension of the underlying physical mechanisms but also provides deep insights into material properties and optimize processing outcomes^6^. Traditional pump-probe technology captures a time slice of information per measurement, reconstructing an ultrafast movie of transient phenomena through repetitive experiments^7–9^. Unfortunately, this approach fails to preserve the authenticity of observation for transient phenomena such as optical rogue waves^10^ and inertial confinement fusion^11^, which exhibit stochastic behavior or difficult to reproduce.

Notably, to overcome the limitations of pump-probe technology, single-shot ultrafast imaging technology encodes time information through different physical dimensions (wavelength/space/polarization) to achieve high-resolution capture of transient phenomena in a single shot. For example, sequentially timed all-optical mapping photography (STAMP)^12,13^ and chirped spectral mapping ultrafast photography (CSMUP)^14,15^ encode time information into the wavelength dimension, subsequently separating the time information using a spatial mapping device and a hyperspectral camera, respectively. Framing imaging based on non-collinear optical parametric amplification (FINCOPA)^16,17^ and frequency-domain integration sequential imaging (FISI)^18^ map temporal profiles to distinct spatial locations via non-collinear optical frequency conversion and multi-angle illumination, respectively. Polarization-resolved ultrafast mapping photography (PUMP)^19^ encodes the time information into the polarization dimension, leveraging a microlens array and a polarization array to disperse temporal information across different regions of the sensor. However, factors such as system complexity, detector size, and the spectral bandwidth of the probe source severely constrain the number of captured frames in such single-shot active framing imaging techniques. Although these techniques possess extremely high-spatial resolution, when an ultrafast phenomenon involves multiple physical mechanisms with different time scales, not only ultra-high-spatial resolution is required, but also high sequence depth (the number of frames in single measurement) is necessary to ensure complete dynamic analysis of transient process.

To avoid losing critical information of transient phenomena within the research time window, single-shot ultrafast compressed imaging^20–23^ has emerged as a potent method by innovatively combining compressed sensing with streak imaging, which enables the acquisition of tens to hundreds of image frames in a single-shot. The core principle of this technique relies on first encoding the spatiotemporal information of the transient phenomenon, O(x, y, t), through modulation by a binary mask. The encoded signal is then dispersed along a one-dimensional spatial axis using a deflection device such as a streak camera^24,25^, time delay integration^26^, galvanometer scanner^27^, diffraction grating^28,29^ or prism^30,31^, producing a projected compressed image on a two-dimensional focal plane array. Finally, the original dynamic scene is computationally reconstructed through compressed sensing algorithms^32–34^. However, since this technique allows spatiotemporal signal aliasing, significant distortion of structural information inconsistent with the projection direction occurs in the reconstructed image. This limitation originates from the one-dimensional spatial-temporal projection used in ultrafast compressed imaging: the spatial frequency analysis of the projected compressed image reveals that the majority of frequency information becomes concentrated along the axis perpendicular to the projection direction. During this process, the complicated multi-directional frequency components present in the original object are modulated into a single-frequency direction, resulting in a serious suppression of spatial frequency intensity along other directions in the transient phenomenon. Such constrained frequency direction-sensing capability makes it extremely challenging to achieve high-spatial-resolution observation of transient phenomena with complex feature information while pursuing high sequence depth. Although numerous excellent works in ultrafast compressed imaging have aimed to improve reconstruction quality^35–40^, they have failed to resolve the key problem of limited frequency direction-sensing capability, resulting in the overall reconstruction quality remaining restricted.

Therefore, the development of new ultrafast imaging techniques that can balance high sequence depth and ultra-high-spatial resolution is an important goal in the field of ultrafast science. In this paper, we show a solution to this problem, proposing a multi-dimensional spatial-temporal projection ultrafast compressed imaging (MSP) method. This approach integrates the multi-angle spatial-temporal data projection module into the traditional single-dimensional spatial-temporal projection system, enabling the acquisition of images with multi-directional projection. Through computational coupling of the projection images from both systems during back-end reconstruction, our method not only effectively restores the transient phenomenon’s information across diverse frequency directions but also achieves significant progress toward higher spatial resolution. Experimental implementation of this approach has yielded both the highest reconstructable lateral and longitudinal resolutions of 813 lp mm^−1^ (corresponding to a linewidth of 620 nm), with an overall omnidirectional spatial resolution exceeding 800 nm for a 60-frame high capture sequence. Furthermore, in experiments involving femtosecond laser-induced side shock wave propagation dynamics and transient surface reflectivity variations, the MSP method successfully captured omnidirectional dynamic details and realized sub-μm-scale precision in dynamic measurements.

## Results

### Principle of MSP

**Fig. 1:**
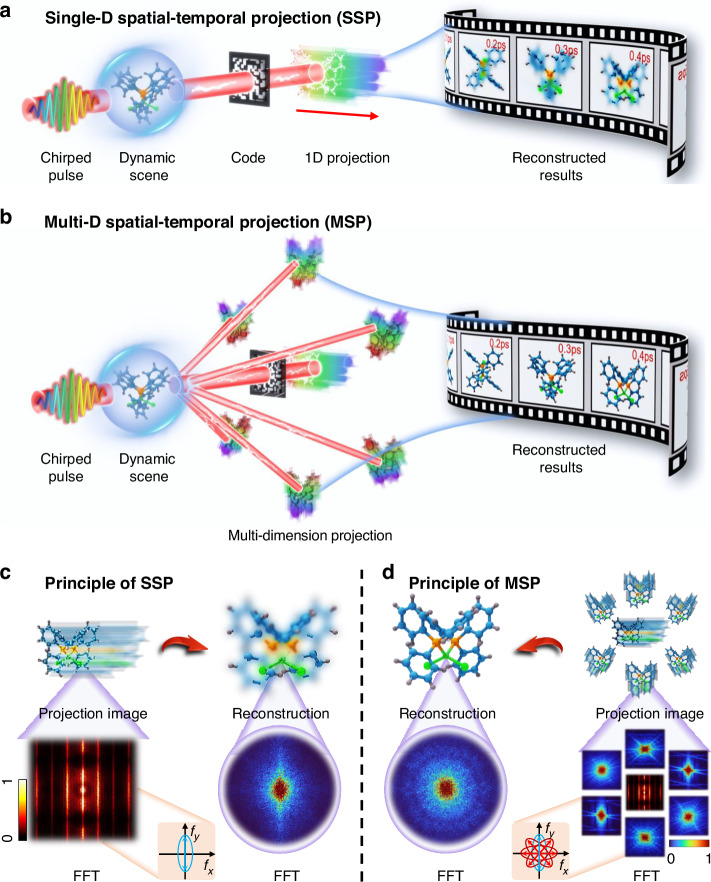
Concept description of multi-dimensional spatiotemporal projection (MSP). **a** Forward model demonstration of single-dimensional spatiotemporal projection (SSP). **b** Forward model demonstration of MSP. **c**, **d** The principle descriptions of SSP and MSP, respectively. (The range of frequency information that can be retained for the corresponding system in the orange box)

The principle concept is shown in Fig. 1. Figure 1a is the forward model of single-dimensional spatial-temporal projection (SSP). A chirped pulse illuminates the dynamic scene. According to the spectral-temporal coupling characteristics, a linear mapping relationship exists between the temporal and spectral information of the dynamic scene^41^. The chirped pulse carrying dynamic information is then spatially encoded by a binary mask, and finally undergoes one-dimensional projection integration through a one-dimensional dispersion element. Since the dynamic process is only projected along a single dimension, the reconstructed images would show obvious directional differences. The reconstruction quality of the object structure aligned with the same or similar to the projection direction is better, while the structural information along other directions is blurred. To address the directional difference in SSP reconstruction results, we integrated a multi-angle projection module into the SSP system, constituting the new multi-dimensional spatiotemporal projection (MSP) system. The forward model of MSP is shown in Fig. 1b. The difference between MSP and SSP is that the chirped pulse carrying dynamic information not only carries out one-dimensional projection, but also passes through the two-dimensional diffraction element to obtain the integral image along multiple projection directions. By coupling multi-directional projection images in the back-end reconstruction, dynamic results with high fidelity in all directions can be reconstructed.

Based on the working principle of SSP, the working principle of MSP is systematically analyzed from both the spatial and frequency domains. As shown in Fig. 1c, one-dimensional projection induces aliasing of spatial and temporal information, resulting in obvious stretching phenomenon within the compressed image. Frequency-domain analysis of the compressed image reveals that the spatial frequency intensity is predominantly concentrated along the axis orthogonal to the projection direction (the inset within the orange box delineates the retained frequency range under one-dimensional projection)^42^. Meanwhile, the reconstruction results also show that the reconstructed structural features are clear in the direction of the same or similar to the projection, while the structural information in other directions is very blurred. This directional difference is further substantiated by Fourier analysis of the reconstructed result, which confirms that single-dimensional projection preserves frequency information solely along a single spatial frequency axis. This method exhibits a significant problem of imbalance multi-directional sensing of spatial resolution, which poses a major challenge to the observation of transient phenomena with complex spatial frequency characteristics.

In contrast to SSP, the MSP system can capture the multi-directional projection images. The Fourier transform of these projection images shows the multi-directional frequency response (Fig. 1d). Consequently, MSP significantly enhances the multi-directional sensing ability of the original data’s frequency information, thereby capturing more comprehensive frequency components (see the orange box for the retained frequency range). It is worth noting that in the process of dynamic imaging, due to the time-varying characteristics of the inter-frame intensity and shape of the transient image, even the integration along the symmetrical direction will produce differentiated frequency characteristics. By using the MSP system, we have successfully realized the ultra-high-resolution reconstruction with balanced spatial frequency sensing capabilities under the condition of high sequence depth acquisition.

### The optical path details of MSP system

**Fig. 2:**
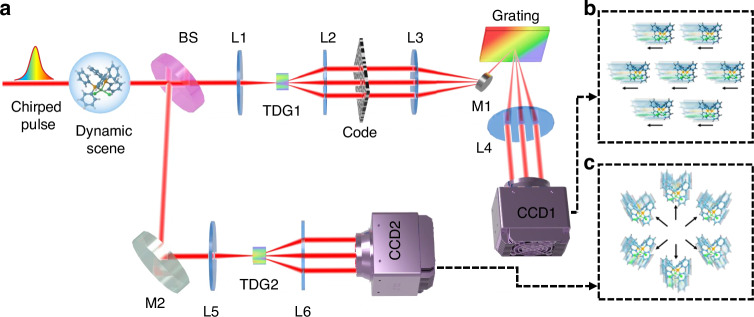
Demonstration of optical path. **a** Details of MSP optical setup. **b** The integral image collected by the single-dimensional high-channel coded projection module. **c** The integral image collected by the multi-angle projection module

As shown in Fig. 2a, a broadened chirped pulse captures spatial-temporal information of dynamic scene. Then, the probe pulse is divided into two paths through the beam splitter (BS). The first path enters the single-dimensional spatial-temporal projection module. The light carrying object information is first mapped on the binary code by the 4 f system composed of L1 (*f* = 50 mm) and L2 (*f* = 50 mm). On the spatial frequency plane, there is a two-dimensional diffraction grating (TDG1) whose internal microstructure can copy the object into 7 copies, and each copy is projected in different regions of the code. In all subsequent experiments, the code used is high-frequency enhanced code. Compared with traditional random code, this code offers a more uniform pixel distribution, preventing the aggregation of white and black pixels when the object is encoded (see Fig. S1 for comparison). Utilizing high-channel encoding imaging can initially obtain acceptable spatial-temporal resolution results. Subsequently, the encoded object is imaged onto CCD1 by a 4 f system consisting of L3 (*f* = 150 mm, achromatic) and L4 (*f* = 150 mm, achromatic). In the middle of L3 and L4, the grating will stretch the spectral-temporal information of the object in one-dimensional direction. Finally, the projection integral image superimposed by different wavelengths is obtained on the CCD (Fig. 2b). Since the dispersion coefficient of the grating is much larger than that of TDG1, the projection directions of different encoding channels in this module are almost the same. It is generally believed that the high-channel coded imaging is a one-dimensional spatial-temporal projection. Another light passing through the beam splitter (BS) enters the multi-angle projection module. The light carrying the same object information is imaged onto the CCD2 through the 4 f system composed of L5 (*f* = 50 mm) and L6 (*f* = 50 mm). There is also a two-dimensional diffraction grating (TDG2) on the spatial spectral plane of the 4 f. However, the function of TDG2 is to disperse and stretch the spectral-temporal information of the object in different directions, resulting in spatial-temporal projection images at 6 uniformly distributed angles within the ring (30°, 90°, 150°, 210°, 270° and 330°), ensuring the capture of omnidirectional spatial frequency (Fig. 2c). These two imaging modules together constitute the experimental system of MSP. After data acquisition, the two captured compressed images are input into a two-step iterative shrinkage/thresholding (TwIST) algorithm based on TV regularization for fusion reconstruction. This algorithm leverages the sparsity of the image in the gradient domain to recover the signal. (Detailed information on MSP reconstruction is provided in Materials and Methods, Supplementary Note 1).

### Simulation

This study first verifies the feasibility of MSP through simulation experiments. In the actual optical setup shown in Fig. 2, MSP employs 7-channel coded imaging combined with 6 projection images across angles, corresponding to 6 projecting directions. Since keeping the number of channels in the simulation consistent with the experimental system makes the simulation results more convincing, the simulation also employs 7 single-dimensional encoding channels and 6 multi-angle projection channels. Totally 40 frames are reconstructed. The SSP system, serving as the control group, adopts 13-channel high-frequency enhanced coded imaging with identical stretching (projection) directions. The simulation samples include static targets with complex spatial frequency characteristics (logos and radial gradient resolution targets) and the dynamic diffusion fringe process to comprehensively evaluate the system performance in spatial resolution and time-varying process reconstruction.

**Fig. 3:**
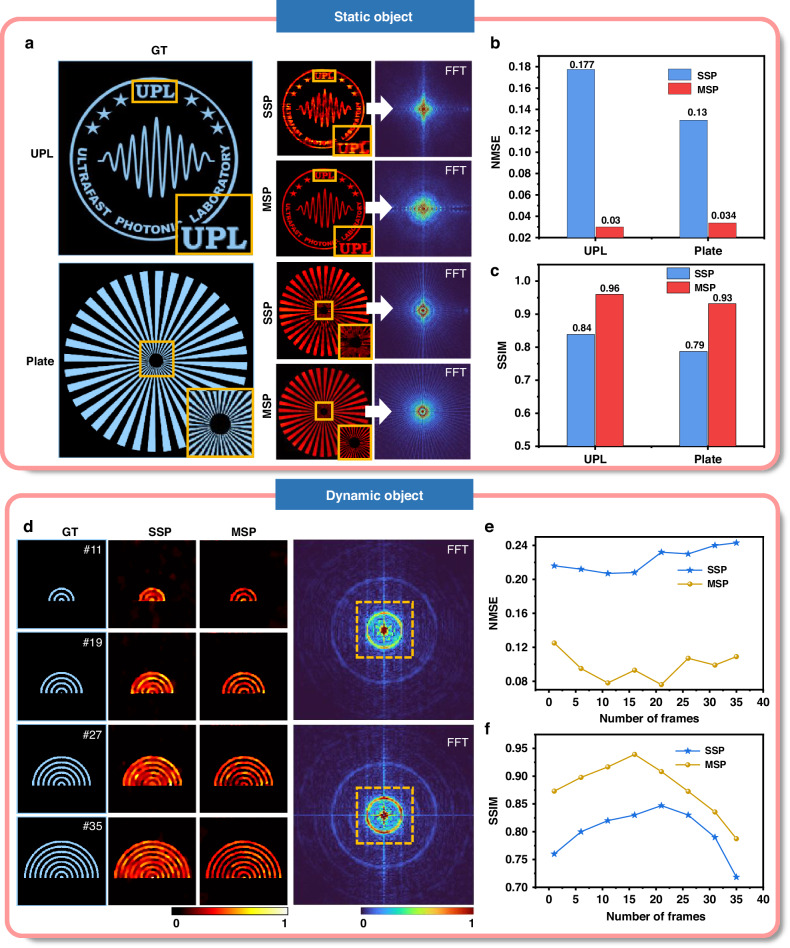
Simulation results. **a** Two groups of static simulation ground truth (GT), the reconstruction results of SSP and MSP system. **b**, **c** The NMSE (Normalized Mean Squared Error) and SSIM (Structural Similarity Index Measure) values calculated according to the data of (**a**). **d** Simulation dynamic process of radial stripe diffusion, the reconstruction results of SSP and MSP systems, the Fourier transform of the 35th frame. **e**, **f** The NMSE and SSIM values of 8 representative frames (take one frame every five frames)

Static reconstruction results demonstrate that MSP exhibits significant advantages in spatial resolution. As shown in the yellow box of Fig. 3a, the logo reconstruction results are highly consistent with the ground truth (GT). Fourier transform analysis reveals that even with an increased number of coding channels (13 channels) to expand the inverse problem-solving conditions, the reconstruction results of the SSP system still exhibit notable frequency sensing discrepancies (longitudinal frequency intensity is much higher than the lateral in the FFT diagram). In contrast, MSP compensates frequency-domain information through integrated spatial frequency multi-dimensional sensing, achieving superior spatial resolution with fewer coding channels. Furthermore, to quantitatively evaluate the reconstruction results, we calculated the NMSE (Normalized Mean Squared Error) and SSIM (Structural Similarity Index Measure) (see Supplementary Note 2). Figure 3b, c visually demonstrate that MSP not only significantly reduces the NMSE by 83% and 73.8%, but also improves the SSIM index by 14.3% and 17.7%. In dynamic process reconstruction, MSP clearly restores the radial diffusion of fringes (Fig. 3d), with all-directional stripes being distinguishable. Comparative experiments indicate that the SSP system can only reconstruct fringes aligned with the stretching (projection) direction clearly, which is limited by single-frequency sensing effects. Moreover, other directional stripes suffer significant quality degradation due to aliasing noise. Frequency analysis of the 35th frame further confirms that MSP significantly enhances all-directional frequency intensities of objects through multi-angle projection, fundamentally improving reconstruction quality. Similarly, we selected eight representative frames (take one frame every five frames) to quantitatively evaluate the dynamic reconstruction results, as shown in Fig. 3e, f. MSP reduces the NMSE by 56.2% on average and improves the SSIM index by 8.9% on average. The computed values still indicate that the MSP exhibits a significant advantage over the SSP, demonstrating the feasibility of the MSP.

### Static performance characterization

This section systematically characterizes the spatial resolution of the MSP system through static experiments. The 13-channel encoded SSP imaging system is employed as the benchmark for comparison. The MSP system retains 7-channel code and incorporated a 6-angle projection integral. Both systems operated under a 60-frame high sequence depth acquisition mode.

**Fig. 4:**
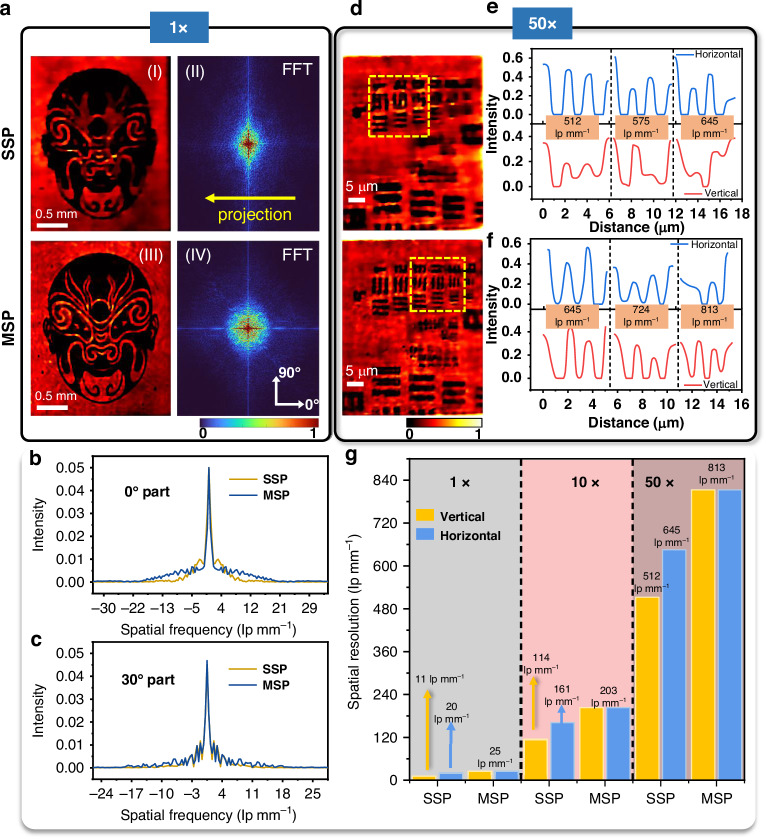
Analysis of static experimental results. **a** At the unit magnification, the reconstruction results of SSP and MSP and the corresponding Fourier transform. **b**, **c** The 0° and 30° intensity curves of the Fourier transform results in (**a**). **d** The highest spatial resolution that SSP and MSP can reconstruct under 50× magnification (the corresponding ground truth in Supplementary Information). **e**, **f** The intensity curves corresponding to the stripes circled by yellow dotted lines in (**d**). **g** At different magnifications, the maximum reconstructable horizontal and vertical resolution of SSP and MSP (see supplementary Fig. S6 for the results of 1× and 10× magnification). The field of view (FOV) in the static experiment is 2.7 mm × 2.2 mm and 54 μm × 43 μm, respectively

To evaluate the multi-dimensional sensing capability, a ‘facial makeup’ with multi-directional spatial frequency features was first selected. As shown in Fig. 4a, although the SSP system significantly increases the number of encoding, its reconstruction performance remains constrained by the single-directional projection mechanism. The Fourier spectrum (II) exhibits distinct directional difference. The frequency intensity perpendicular to the projection direction is dominant, resulting in superior identification of horizontal structures over other orientations in the reconstructed image. In contrast, the MSP system effectively achieves frequency equalization through multi-angle projection integration. Both the reconstructed image and Fourier spectrum (IV) enabling demonstrate clear visualization of features in all orientations of ‘facial makeup’. To more clearly characterize this feature, intensity profiles along 0° (vertical structure in spatial domain) and 30° were extracted from (II) and (IV), as shown in Fig. 4b, c. The frequency response in the 0° direction reflects the key difference (Fig. 4b): The resolvable cutoff frequency of SSP is 8.47 lp mm^−1^. However, the MSP is 17.84 lp mm^−1^, which is more than double that of the SSP. This improvement stems from the fact that MSP has the projection integral in this direction, capturing the corresponding frequency information during the data acquisition stage. Therefore, its reconstruction capability is significantly higher than that of SSP. Similarly, as shown in the 30° direction plot (Fig. 4c), SSP exhibits a cutoff frequency of 9.65 lp mm^−1^, while MSP achieves 17.75 lp mm^−1^, leading to the same conclusion. Combining the reconstructed images and quantitative analysis, it can be concluded that the MSP system effectively restores omnidirectional frequency information under high sequence depth conditions.

To investigate the limit resolution achievable by the MSP imaging system at 60 frames, the resolution test chart was imaged at 50× magnification. The comparison results in Fig. 4d show that the reconstruction results of MSP are closer to the ground truth in terms of spatial fidelity compared with the reconstructed images of SSP, and show better contrast performance for narrow-linewidth fringes. By extracting the intensity distribution of horizontal and vertical stripes (SSP reconstruction data taken from Group 9, Element 1–3; MSP data from Group 9, Element 3–5), Fig. 4e reveals that SSP achieves a resolution of 645 lp mm^−1^ in the horizontal direction, while the vertical resolution is only 512 lp mm^−1^, exhibiting significant directional differences characteristics. In contrast, the MSP system improves resolution to 813 lp mm^−1^ (Fig. 4f) which is benefited by its multi-dimensional projection mechanism. It should be noted that although the imaging quality of the horizontal stripes at Group 9, Element 5 of the ground truth is limited by the quality of resolution test chart, the stripes in the reconstructed image are highly similar to the ground truth (see supplementary Fig. S5). It can be considered that MSP system achieves a spatial resolution of 813 lp mm^-1^ in both vertical and horizontal directions. In the comparison with the GT image, the NMSE values for the SSP and MSP reconstruction results are 0.23 and 0.12, respectively, which further indicates that the reconstruction results of MSP are very close to GT images. In addition, the supplementary Fig. S4 lists 10 reconstructed representative frames of ‘facial makeup’ and a resolution test chart (displaying one frame every 6 frames). Although the 60-frame reconstructions exhibit spectral dependence, the reconstruction resolution of MSP is better than 800 nm for all reconstructed images. Finally, we compared the highest spatial resolution characteristics of the two imaging systems at different magnification levels (1×, 10×, 50×). Figure 4g shows that the SSP system consistently achieves better resolution in the horizontal direction than in the vertical direction. Moreover, this multi-dimensional sensing disparity persists across all tested magnification levels. The MSP system achieves isotropic resolutions of 25 lp mm^−1^, 203 lp mm^−1^, and 813 lp mm^−1^ at different magnification levels. Compared to the SSP system, it improves lateral resolution by about 25% and longitudinal resolution by about 59%–127%, demonstrating outstanding omnidirectional spatial resolution capability.

### Femtosecond laser-induced ultrafast shock wave expansion

**Fig. 5:**
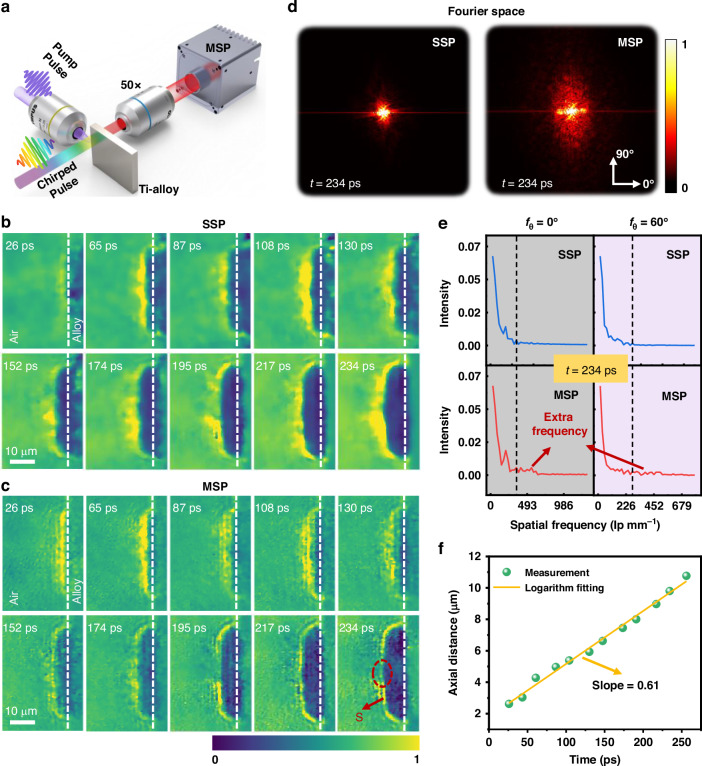
Femtosecond laser-induced ultrafast shock wave expansion. **a** The optical setup of the experiment. **b**, **c** The reconstruction results of SSP and MSP (The size of the original image is 760 × 620. To better display, the size of the image here has been cut, and the size is 700×490. The original FOV is 52 μm × 43 μm). **d** The Fourier transform of the reconstruction result at *t* = 234 ps. **e** The extracted intensity curves along the 0° and 60° directions from (**d**). **f** According to the reconstruction result of MSP, the extracted curve of the axial distance of the shock wave with time

Femtosecond laser-induced ultrafast shock wave expansion dynamics holds critical research significance in nonlinear and non-equilibrium processes such as microchannel fabrication^43,44^, laser shock peening^45^, and laser-induced breakdown spectroscopy^46^. The transient evolution of shock waves can reveal the physical mechanisms underlying laser–matter interactions. In this section, the dynamic reconstruction capability of MSP is validated through side-view observations of shock waves induced by femtosecond laser irradiation on the titanium alloy surface, the experimental setup of which is illustrated in Fig. 5a (see the Method for details). The experiment adopted a dynamic observation window of 256 ps and achieved the precise capture of 60 continuous transient images under the condition of a single pulse energy of 30μJ.

Figure 5b, c present the reconstructed dynamic evolution of laser-induced shock waves in the air in front of the sample using the SSP and MSP methods, respectively, where the ratio of excitation images to background images was used to enhance contrast. The dashed lines in the images indicate the sample-air interface. Ten representative frames from the dynamic process are displayed to illustrate the spatiotemporal evolution (the complete ultrafast dynamics are provided in Supplementary movie S1). With the continuous deposition of laser energy in the material, the surface of the material will be partially melted or even vaporized in a very short period of time, resulting in plasma clusters. These plasma clusters exhibit characteristic a dark non-transparent region in transient images due to their strong absorption of the probe pulse^47^. Notably, the bright fringes surrounding the plasma region arise from refractive index gradients between the ionized zone and ambient air^48,49^. Moreover, these fringes can be seen in the reconstruction results of MSP but cannot be identified in the results of SSP. Over time, the ejection of materials from the surface induces variations in air temperature and pressure, resulting in observable continuous expansion of the shock wave front within the observation window.

A comparison between Fig. 5b, c reveals that MSP not only reconstructs the shock front (S) but also resolves a subtle convex structure at its center. This phenomenon can be attributed to the interaction between the pump laser-induced ionization channel in air and the expanding shock wave, where localized pressure reduction accelerates the shock velocity, manifesting as the small protrusion at the shock wave front^50^. Figure 5d displays the Fourier transform image at *t* = 234 ps (55th frame), demonstrating that the MSP system, with its multi-angle projection capability, achieves a broader frequency reconstruction range. To quantitatively compare the imaging quality, frequency intensity profiles at 0° and 60° orientations are extracted from Fig. 5d. As shown in Fig. 5e, the dashed black line marks the highest frequency resolvable by SSP, while MSP surpasses this limit, achieving submicron-scale dynamic resolution at higher frequencies. Additionally, to demonstrate that the extra frequency components in MSP shown in Fig. 5e correspond to actual signal values, pump-probe images at the corresponding time points were captured (see Fig. S7(a)). As a reference, the reconstruction results of MSP are closer to the pump-probe images. Furthermore, the corresponding Fourier spectra reveal that the extra frequency components in the MSP curve correspond to detailed features of the transient phenomenon, reflecting actual signal frequency intensity (see Fig. S7(b) and Fig. S7(c)). Finally, based on the MSP reconstruction in Fig. 5c, the shock wave expansion characteristics of femtosecond laser-irradiated titanium alloy are analyzed. By tracking the axial distance from the shock wave front to the material surface, a linear increase in axial distance over time is observed within the observation window. The yellow line in Fig. 5f represents a double-logarithmic fit^51,52^, yielding a slope of 0.61, proportional to *t*^2/3^, consistent with one-dimensional expansion behavior predicted by the Sedov-Taylor theory^53^ (see Supplementary Note 4). These results align well with the experimentally observed dynamics.

### Ultrafast evolution of silicon surface ablated by femtosecond laser

**Fig. 6:**
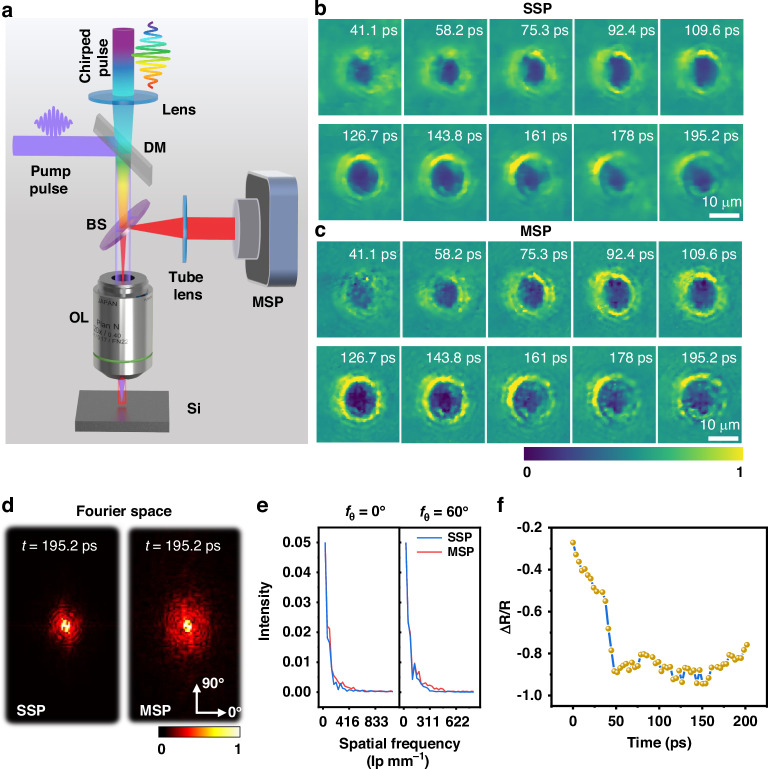
Ultrafast evolution of silicon surface ablated by femtosecond laser. **a** The optical setup of the experiment. **b**, **c** The reconstruction results of SSP and MSP (The size of the original image is 760 × 620. To better display, the size of the image here has been cut and the size is 580 × 500. The original FOV is 52 μm × 43 μm). **d** The Fourier transform of the reconstruction results at *t* = 195.2 ps. **e** The extracted intensity curves along the 0° and 60° directions from (**d**). **f** The extracted curve of the relative reflectivity of the silicon surface with time according to the reconstruction results of MSP

The visualization of phenomena involved in femtosecond laser ablation of material surfaces provides critical insights into processes such as plasma formation, material melting. In this section, we employ the MSP system to observe the ultrafast evolution of femtosecond laser ablation on the silicon surface with 60 frames and time window of 202 ps. The optical configuration is illustrated in Fig. 6a (see the Method for details). Figure 6b, c present the reconstruction results from SSP and MSP, respectively. Similar to the previous experiment, this experiment also utilized the ratio of excitation images to background images were used to enhance contrast. Ten representative frames selected to illustrate the dynamic evolution (full ultrafast dynamic imaging results are provided in Supplementary movie S2). The images reveal that when the pump pulse is focused onto the silicon surface, a dark region emerges at the ablation center, and its area expands over time. Concurrently, the reflectivity of this region decreases. After approximately 100 ps, ring-shaped patterns become distinctly observable in the MSP reconstruction. These rings are Newton ‘s rings formed by the interference pattern generated by the partial reflection of the ablation layer and the reflection of the remaining surface below after the silicon is irradiated by the laser^54,55^. The shape of these rings corresponds to multi-directional frequency components in the spatial spectrum. Figure 6d shows the Fourier transform image of the reconstruction results at *t* = 195.2 ps, while Fig. 6e presents the extracted frequency intensity profiles from the FFT images for quantitative characterization. The results demonstrate that, compared to SSP, MSP significantly improves the identifiable range of the cutoff frequency and successfully recovers the frequency information in the non-projection direction. Similarly, we compared the results of SSP, MSP, and pump-probe imaging (see Fig. S8(a)). MSP can identify the Newton’s rings generated by ablation, aligning more closely with the reconstruction results from pump-probe imaging. Furthermore, the presented FFT images and extracted frequency intensity curves once again demonstrate the dynamic reconstruction capability of MSP (see Fig. S8(b) and Fig. S8(c)). Finally, the temporal evolution of the surface relative reflectivity was analyzed based on MSP reconstructions, as shown in Fig. 6f. At early stages, the surface gradually absorbs laser energy, leading to melting and a corresponding decline in relative reflectivity. The reflectivity reaches its minimum at approximately 50 ps. Subsequently, as the pump laser induces strong electronic excitation, the surface transitions into an extremely hot, pressurized fluid state^56^. The ablated region remains at elevated temperatures, exhibiting high absorption and low reflectivity, thereby stabilizing the relative reflectivity. Minor fluctuations in the curve may originate from spatial inhomogeneity of the probe beam and spectral intensity variations of the chirped pulse. These microscopic transient observations further validate the superior performance of spatial frequency multi-dimensional sensing ultrafast compressed imaging in resolving ultrafast dynamics.

## Discussion

In general, we use the multi-dimensional spatial-temporal projection ultrafast compressed imaging method to achieve accurate observation of microscopic transient phenomena with large frames and extremely high-spatial resolution. Simulation experiments demonstrated that the NMSE (reduced by 56%-80%) and SSIM (increased by 8.9%-14%) of reconstruction results have been significantly improved. Similarly, quantitative analysis in static experiments shows that MSP enhanced omnidirectional spatial resolution, with the highest resolution reaching 620 nm. Furthermore, during the transient process of observing femtosecond laser ablation of materials, visualization images and frequency curve analysis of the lateral shock wave expansion and surface reflectivity changes confirmed that MSP can capture finer information at sub‑micron dynamic resolution. Therefore, whether in static or dynamic experiments, it is precisely because of the addition of multi-angle projection integral images that the frequency information in different directions of the image is obtained, which enables MSP to have higher reconstruction fidelity. This imaging method improves the anisotropic reconstruction of traditional ultrafast compressed imaging and is a powerful tool for observing ultrafast complex phenomena.

To capture ultrafast phenomena with rich feature structures, a long time window on the order of hundreds of picoseconds was selected as the probe temporal width in this study and the frame rates calculated accordingly are 0.23 THz and 0.3 THz. It should be noted that the temporal and spectral width of the chirped probe light source play a decisive role in the temporal performance of the MSP system. According to descriptions of other studies using similar chirped pulse, the theoretical limit of temporal resolution can be relaxed when the spectral phase of the probe pulse can be retrieved^30,57^. When the probe pulse duration is 40 fs with a spectral width of 80 nm, a temporal resolution can reach tens of femtoseconds^30^ (corresponding to the Fourier transform limit).

As a computational imaging technique based on compressed sensing, the reconstruction process utilizes a two-step iterative shrinkage/thresholding (TwIST) algorithm based on TV regularization has significant advantages in solving the inverse problem of high-channel imaging^35^. On a computer equipped with an Intel(R) Core (TM) i7-10700 CPU @ 2.90 GHz using MATLAB R2020b, the computational time for reconstructing a data cube of size 760 × 620 × 60 is approximately 15 minutes. Compared with the running time of other single-shot compressed imaging^58^, this time reaches the average level, which is acceptable for post‑processing analysis.

The core idea of MSP is the introduction of multi-angle projection, which extends sparse-view computational tomography to the field of ultrafast imaging. Li et al. proposed single-shot frequency domain tomography (SS-FDT)^59^, which captures phase projections of the target object from multiple angles in ultrafast scenarios and employs computational tomography reconstruction algorithms to generate dynamic image sequences, laying the foundation for ultrafast dynamic computational tomography imaging. Additionally, it is worth mentioning that in recent years, some novel ultrafast compressed imaging techniques, though not specifically aimed at improving reconstruction quality, have made significant contributions to the isotropic reconstruction of images. For example, the swept coded aperture real-time femtophotography (SCARF)^29^ proposed by Liu et al. This work is to introduce space-time shearing through the dispersion of the grating before encoding the object, and then each frame of the transient phenomenon corresponds to a different code. Finally, the shear effect introduced before is eliminated by the reverse dispersion of the second grating to obtain the integral image. The advantage of this method is that there is no stripe structure formed by one-dimensional shearing in the integral image. The directionality of the spatial resolution in the reconstructed image is guaranteed. However, this method requires pre-shearing of the ultrafast process, which is not suitable for the passive detecting of ultrafast dynamics through streak camera. In passive imaging, the MSP concept enables multi-dimensional spatial-temporal projections using multiple Dove prisms, without the need for preprocessing the target signal. Compressed ultrafast tomographic imaging by passive spatiotemporal projections (CUTI)^60,61^ proposed by Lai et al. achieves the acquisition of multiple spatiotemporal projections of three-dimensional data (x, y, t) by varying the scanning speed of the streak camera. However, this technique can only capture one projection at a time, requiring multiple shots to complete the measurement. Additionally, the technique relies on the scanning direction of the streak camera, limiting the acquisition of projection data to specific orientations. Single-shot ultrafast imaging based on round-view projection (RVP)^62^ proposed by Lu et al., which realizes the quasi-omnidirectional projection integral of the spatial-temporal data cube and obtains better reconstruction fidelity. However, the number of captured frames of this technique is limited by the shear speed of the two-dimensional diffraction grating (only 22 frames), so it is necessary to broaden the spectral width of the chirped light to increase the number of frames. MSP integrates multi-angle spatial-temporal projection into traditional ultrafast compressed imaging based on grating one-dimensional projection, enabling both the ability to capture large frames in a single-shot and achieve ultra-high-spatial resolution. The comparison of MSP to several representative techniques in single-shot ultrafast imaging techniques with high-spatial resolution is summarized in Supplementary Table 1.

Although MSP significantly enhances omnidirectional spatial resolution, certain trade-offs and limitations must be considered. First, the system requires the use of a two-dimensional diffraction grating to split the beam for high-channel encoding and multi-angle projection, which reduces the photon efficiency per projection channel. Consequently, higher detection energy or longer exposure times are needed to capture weak-light phenomena. For ultrafast phenomena with specific structural distribution directions, such as laser-induced plasma, the projection direction of interest can be selected as needed to improve energy utilization efficiency. Second, the current number of captured frames is limited by the employed spectral bandwidth (20.4 nm in dynamic experiments). This problem could be addressed by increasing the spectral width or utilizing a supercontinuum light source, enabling the extension of frame counts to over 100 frames without sacrificing temporal resolution. Third, in the reconstruction coupling process, the prior matrix of the multi-angle projection module in the coupling reconstruction process is theoretically a full 1 matrix. However, in the experimental calibration process, the intensity distribution of the illumination beam is regarded as the prior matrix of this part. Therefore, the quality of the illumination beam seriously affects the quality of the reconstruction, and some noise can be found in the background of the reconstructed image. Since the probe light passes through multiple optical components during transmission, both the propagation distance and the surface quality of the components inevitably degrade the probe light quality. Non‑uniform illumination will attenuate high‑frequency components in low‑intensity regions, which manifests as reduced contrast between the low‑frequency background and image details in reconstructions. To mitigate this problem, a spatial filtering module could be added before the light enters the MSP imaging system. This would yield a prior matrix with more uniform intensity distribution and a cleaner background during experimental calibration. Additionally, although the currently used TwIST algorithm is suitable for solving high‑channel imaging problems, it remains a conventional optimization‑based reconstruction framework. When substantial noise is present in actual measurements, or the signal itself is not perfectly sparse, the algorithm tends to cause detail blurring. Currently, plug-and-play (PnP) algorithms^63^ have been widely used to embed deep learning pre-trained denoising networks into the regularization terms of traditional optimization models. Moreover, researchers have already applied such models to high‑channel imaging and achieved excellent reconstruction quality^38,64^. In the future development of the MSP system, we also intend to leverage the PnP framework to develop reconstruction algorithms tailored to the current system, thereby enabling further breakthroughs in spatial resolution.

Finally, MSP holds broad application prospects in the field of ultrafast science. The ultrafast mechanisms of femtosecond laser–matter interaction investigated in this work can be extended to micro‑nano processing phenomena in various materials. For example, the fabrication of photonic chips involves multiple nonlinear effects. Using MSP to observe transient refractive index changes could facilitate the creation of three‑dimensional optical waveguides inside such materials, enabling true three‑dimensional photonic integration^65^. Meanwhile, MSP has also opened promising path for studying ultrafast processes in other complex, non‑repeatable systems. Many critical high‑speed biological events with complex structural features (such as the propagation of neuronal action potentials^25,66^) can be investigated using MSP’s comprehensive capabilities of omnidirectional high‑fidelity imaging and high sequence depth, thereby providing insights into the mechanisms underlying physiological and pathological states. In addition, coupling long‑time‑window probe light^67,68^ with MSP or applying the concept of MSP to passive ultrafast imaging could enable the study of physical or biological transient phenomena occurring on nanosecond or longer timescales.

## Materials and methods

### The experimental setup of femtosecond laser-induced ultrafast shock wave

The output 800 nm femtosecond laser is frequency-doubled via a nonlinear crystal to generate 400 nm wavelength, subsequently focused onto the titanium alloy surface through a 10× objective lens. A synchronously triggered 800 nm chirped probe pulse captures this ultrafast process from the lateral direction, magnified by a 50× objective lens, and ultimately imaged onto the MSP system.

### The experimental setup of silicon surface ablated by femtosecond laser

400 nm, 7 μJ pump pulse is reflected by a dichroic mirror (DM) and focused onto the silicon surface using an objective lens (Nikon, 20×, NA = 0.45). A 202 ps linearly chirped probe pulse is transmitted through the DM, collinearly illuminates the ablated region. Through the reflection mode, the captured transient phenomenon is imaged into MSP by a 4 f system composed of an objective lens and a tube lens (f = 600 mm). Finally, the dynamic evolution of the ablation process was recorded with 60 frames of ultrafast sequence images.

### The forward model and reconstruction of MSP

In the process of collecting the three-dimensional data of the transient phenomenon ***O***(x, y, t), MSP will use two cameras to collect two different sets of two-dimensional compressed images. The first camera is used to capture encoded single-dimensional spatial-temporal projection (SSP) data, and the second camera is used to capture uncoded multi-angle projection data. Throughout the experiment, the imaging system has a unit magnification, and the two cameras have the same pixel size. In our system, the transient phenomenon is first illuminated by a chirped pulse to convert the time information into spectral-temporal information. This process can be expressed as operator ***I***.

$${{\boldsymbol{O}}}^{{\boldsymbol{{\prime} }}}(x,y,\lambda )={\boldsymbol{I}}\{{\boldsymbol{O}}(x,y,t)\}$$

For SSP, we use the high-channel spectral-temporal active recording device. The transient phenomenon after time-frequency transformation is encoded by multiple high-frequency enhanced masks, which is denoted by the operator ${{\boldsymbol{C}}}_{{\boldsymbol{i}}}$(*i* = 1, 2, …, 7). Subsequently, the encoded transient phenomenon is dispersed and stretched by the grating along the one-dimensional space direction, which is denoted by the operator ***M***. Finally, a 2D camera records the integral data ${{\boldsymbol{E}}}_{{\boldsymbol{i}}}$(x, y) of one-dimensional dispersion projection, which is denoted by the operator ***T***. In summary, the forward model of SSP can be expressed as:

$$\left[\begin{array}{l}{E}_{1}\\ {E}_{2}\\ \vdots \\ {E}_{7}\end{array}\right]=\left[\begin{array}{l}\begin{array}{l}TM{C}_{1}\\ TM{C}_{2}\\ \vdots \end{array}\\ TM{C}_{7}\end{array}\right]O\mathrm{'}(x,y,\lambda )$$

For multi-angle projection, the system does not use binary mask. The transient phenomenon after time-frequency transformation is directly projected and stretched by a two-dimensional diffraction grating along multiple dimensions in space, which is denoted by the operator ${{{\boldsymbol{M}}}^{{\boldsymbol{{\prime} }}}}_{{\boldsymbol{i}}}$(***i*** = 1,2, …,6). Finally, a 2D camera, which is the same as capturing the SSP, is used to record the integral data ${{{\boldsymbol{E}}}^{{\boldsymbol{{\prime} }}}}_{{\boldsymbol{i}}}$(x, y) of the multi-angle projection. This process is denoted by the operator ***T***. In summary, the forward model of multi-angle projection can be expressed as:

$$\left[\begin{array}{l}\begin{array}{l}{{E}^{{\prime} }}_{1}\\ {{E}^{{\prime} }}_{2}\\ \vdots \end{array}\\ {{E}^{\mathrm{'}}}_{6}\end{array}\right]=\left[\begin{array}{l}T{{M}^{{\prime} }}_{1}\\ T{{M}^{{\prime} }}_{2}\\ \vdots \\ T{{M}^{\mathrm{'}}}_{6}\end{array}\right]{O}^{\mathrm{'}}(x,y,\lambda )$$

These two formulas can be briefly expressed as

$${E}_{i}={S}_{i}O$$

When given the operator ***S*** and the measurement matrix ***E***, we employ the TwIST algorithm to iteratively optimize the following objective function, thereby solving the ill-posed inverse problem in Eq. (4):

$$\mathop{\mathrm{arg}\,\min }\limits_{o}\left\{\frac{1}{2}||E-SO|{|}_{2}^{2}+\lambda \Phi (O)\right\}$$

Here, $\frac{1}{2}{||E}-{SO}{{||}}_{2}^{2}$ is the fidelity term, Φ(O) is the regularization term, and *λ* is the regularization parameter to balance the fidelity term and the regularization term. See supplementary Note 1 for details on the reconstruction algorithm.

## Supplementary information

Supplementary

Movie S1

Movie S2

## Data Availability

The authors declare that all data supporting the findings of this study can be found within the paper and its Supplementary Information files. Additional data supporting the findings of this study are available from the corresponding authors upon reasonable request.
