# Supplementary material for: Multi-dimensional spatial-temporal projection ultrafast compressed imaging
Source: Light Sci Appl. 2026 Jun 29;15:294. doi: 10.1038/s41377-026-02317-2 (PMC13314981; doi:10.1038/s41377-026-02317-2)
Supplement: Supplementary file 1 — Supplementary [file 41377_2026_2317_MOESM1_ESM.pdf]

Supplementary Information for

Multi-dimensional Spatial-temporal Projection Ultrafast Compressed Imaging

Yizhao Meng[1], Pengfei Zhang[1], Jiaxin Yin[1], Yu Lu[1, *], Kaiduan Yue[2], Fei Yin[2, 3], Qing Yang[2], and Feng Chen[1, *]

[1] *State Key Laboratory for Manufacturing System Engineering and Shaanxi Key Laboratory of Photonics Technology for Information, School of Electronic Science and Engineering, Xi'an Jiaotong University, Xi'an, 710049, China*

[2] *School of Instrument Science and technology, Xi'an Jiaotong University, Xi'an, 710049, China*

[3] *Xi'an Institute of Optics and Precision Mechanics (XIOPM), Chinese Academy of Sciences (CAS), Xi'an 710119, China*

[*] chenfeng@mail.xjtu.edu.cn, luyu90@xjtu.edu.cn

Note 1: Details of MSP's reconstruction process

Before entering the back-end reconstruction, we will calibrate the linear operators $\boldsymbol{S_i}$ and $\boldsymbol{S'_i}$ in advance. In this paper, we use the two-step iterative shrinkage/thresholding (TwIST) algorithm [1] to minimize the objective function to solve the inverse problems of Eq. (2) and (3) to restore the transient phenomenon:

$$f(\hat{o})_{TwIST} = \underset{O}{\operatorname{argmin}}\left\{\frac{1}{2}\|E - SO\|_2^2 + \lambda\Phi(O)\right\} \tag{S1}$$

Where $E = [E_i,\ \tau E'_i]^{\mathrm{T}}$ and $S = [TMC_iI,\ \tau TM'_iI]^{\mathrm{T}}$ are the compressed data captured by two cameras and the prior matrices of each operator obtained by pre-calibration, respectively. τ is the weight factor to balance the light intensity of the two imaging systems by adjusting the energy in the system. $\frac{1}{2}\|E - SO\|_2^2$ is the fidelity term, Φ(O) is the regularization term, and λ is the regularization parameter to balance the fidelity term and the regularization term. The regularization function Φ(O) is the total variation (TV) [2], defined as:

$$\Phi_{TV}(O) = \sum_{m=0}^{N_t-1}\sum_{i,j}\left\|D_{i,j}O_m\right\|_2 + \sum_{n=1}^{N_x-1}\sum_{i,j}\left\|D_{i,j}O_n\right\|_2 + \sum_{p=1}^{N_y-1}\sum_{i,j}\left\|D_{i,j}O_p\right\|_2 \tag{S2}$$

Where $D_{i,j}$ is the discrete gradient calculation of scene O in the horizontal i and vertical j directions, n, p, m represents the discrete index of scene O in the x, y, t dimensions, and N_x, N_y, N_t represents the discrete size of scene O in the x, y, t dimensions.

Before performing image fusion, we first need to determine the relative positions

among different channels. Using the calibration optical path described in Supplementary Note 3, we capture encoded images for each frame contained in all channels. Subsequently, a specific frame from one channel is selected as the reference frame, and cross-correlation is performed between this reference frame and the images from other channels to determine the relative coordinates of each image. Then, by inputting the obtained prior matrix and compressed images into the multi-channel fusion step of the reconstruction algorithm, these frames are inversely shifted to the positions corresponding to the reference frame to achieve the final image fusion reconstruction.

Note 2: Quantitative characterization of simulation results

To quantitatively characterize the reconstruction quality of the simulation experiment, we use **NMSE** (Normalized Mean Squared Error) and **SSIM** (Structural Similarity Index Measure) two indicators. NMSE is an indicator used to measure the prediction error of the model. It normalizes the mean square error (MSE) so that data of different scales or dimensions can be compared. Its calculation formula is:

$$\text{NMSE} = \frac{\sum_j \left(X_j - X_j^*\right)^2}{\sum_j \left(X_j\right)^2} \tag{S3}$$

Where X_j is the original signal and X_j^* is the reconstructed signal. It can be seen from the formula that the closer the **NMSE** is to 0, the better the reconstruction is.

SSIM is an index to measure the similarity of two images. It comprehensively measures the similarity of two images from three aspects: brightness, contrast and structure. Its calculation formula is:

$$\text{SSIM}(x, y) = \frac{\left(2\mu_x\mu_y + C_1\right)\left(2\sigma_{xy} + C_2\right)}{\left(\mu_x^2 + \mu_y^2 + C_1\right)\left(\sigma_x^2 + \sigma_y^2 + C_2\right)} \tag{S4}$$

Where μ is the mean value of the calculated image, σ_{xy} is the covariance of the two images, σ^2 is the variance of the calculated image, and C is the stability constant. The closer the **SSIM** is to 1, the better the reconstruction quality is.

The results of quantitative characterization according to the formula are shown in Fig.

3.

To characterize the situation that MSP reduces the reconstruction blur at the omnidirectional angle, the image differential value at different angles is calculated by using the 35th reconstruction frame of the dynamic simulation experiment. The calculation formula of differential is as follows:

$$dx(i,j) = I(i+1,j) - I(i,j) \tag{S5}$$

$$dy(i,j) = I(i,j+1) - I(i,j) \tag{S6}$$

I is the pixel value of the image, i and j are the coordinates of the pixels. In this calculation, the angle range is selected as 0-180 °, and the differential value is calculated every 10 °. The differential value calculation formula at different angles is as follows:

$$D(\theta) = dx * \cos(\theta) + dy * \sin(\theta) \tag{S7}$$

Then, the squared sum of the differential matrices at each angle are calculated to obtain a total energy value:

$$E(\theta) = \sum_{i,j} D(\theta)^2 \tag{S8}$$

When the pixel values of an image change relatively smoothly, the calculated total energy value will be lower. When there are sudden changes or obvious edges in the image, the calculated total energy will be larger. As shown in the Fig S2, for SSP, the total differential energy of is notably high only around 90°, which indicates that the SSP can only reconstruct the horizontal stripes with better contrast. In other directions, especially at 0° or 180°, the total energy is very low, indicating that smooth pixel variations in those directions, that is, corresponding to the blurred image. This phenomenon occurs precisely because SSP lacks projection images in those directions, resulting in the suppression of frequency information along those directions. In contrast, when analyzing the angular variation of differential energy for MSP, we observe that MSP maintains a relatively high energy across all angles, which is consistent with the change trend of the ground truth. This phenomenon directly results from the inclusion of multi-angle projections in MSP, which captures frequency information from multiple directions. Consequently, MSP effectively mitigates the blurring and aliasing present in SSP, yielding high-quality reconstruction results.

Note 3: The calibration details of MSP's spatial coding

In the reconstruction process of the previous section, it is mentioned that the linear operators $\boldsymbol{S_i}$ and $\boldsymbol{S'_i}$ need to be obtained in advance before entering the back-end reconstruction. $\boldsymbol{S_i}$ and $\boldsymbol{S'_i}$ are actually obtained by spatial coding after a series of linear transformations. Due to various aberrations, noise and the influence of the spectral intensity of the femtosecond laser itself in the imaging process, there is a big difference between the actual spatial coding image and the theoretically designed coding matrix. Therefore, it is necessary to recalibrate to obtain a spatial coding image that can be used for reconstruction.

As shown in Fig. S3a, we have a spectral selection module before the imaging system. A femtosecond laser is first spatially dispersed by a grating (G1), and the lens (L1, f=80 mm) gathers the dispersed light into a line on the focal plane. Each position on this line represents the different wavelengths. On this plane, we place a slit with adjustable width. By adjusting the width of the slit, we can select femtosecond pulses with different spectral widths. The lens (L2, f=80 mm) and grating (G2) reconvert the selected spectrum into femtosecond pulses without spatial dispersion.

First of all, in the MSP imaging system, we only place the spatial encoded pattern that has been etched in the corresponding position. Subsequently, we narrowed the slit so that a clear coded pattern can be observed on the CCD (Fig. S3b). At the position of the slit, we add a precision translation stage. By changing the position of the slit on the focal plane, we can obtain the coded patterns corresponding to different wavelengths. Fig. S3c shows the linear operator $\boldsymbol{S_i}$ of the SSP system obtained by the calibration system, and Fig. S2d shows the linear operator $\boldsymbol{S'_i}$ of the MSP system obtained by the calibration system (only one of the projection dimensions is shown).

Note 4: Sedov-Taylor theory

The side shock wave induced by femtosecond laser can be approximated as the result of instantaneous massless point explosion. The propagation of shock wave can be described by Sedov-Taylor theory [3]:

$$\mathrm{R} = \lambda \left(\frac{E}{\rho}\right)^{1/(\beta+2)} t^{2/(\beta+2)} \tag{S9}$$

Where R is the expansion distance, λ is a constant related to the specific heat capacity, approximately in units, E is the energy converted into the plasma state, ρ is the density of the unperturbed air, and t is the propagation time. β denotes the dimension of propagation: for spherical propagation $\beta = 3$, for cylindrical propagation $\beta = 2$, for plane propagation $\beta = 1$.

In the calculation, the logarithm of both sides of Eq. (S8) is taken at the same time:

$$\mathrm{lnR} = \frac{2}{\beta + 2}\mathrm{ln}t + \mathrm{ln}\left(\lambda \left(\frac{E}{\rho}\right)^{1/(\beta+2)}\right) \tag{S10}$$

It can be seen from the Eq. (S7) that ln$R$ is linearly related to ln$t$. According to the variation of the axial distance with time drawn in Fig. 4f, the slope of the logarithmic fitting is calculated to be 0.61, that is, $2/(\beta + 2)$=0.61. Therefore, β = 1.27, the propagation of the shock wave belongs to plane propagation, which is consistent with the observed results.

Note 5: Performance of MSP

Sequence depth

The number of frames that can be captured by the MSP system is only related to the one-dimensional dispersion grating. The dispersion parameters can be expressed as:

$$\alpha = \frac{f}{a \cdot cos\varphi} \tag{S11}$$

Here, f is the focal length of the lens L4, a is the grating period number, and φ is the diffraction angle.

According to the dispersion parameters, the wavelength interval of adjacent frames can be expressed as:

$$\delta\lambda = \frac{l}{\alpha} \tag{S12}$$

l is the spatial distance between each frame. In the dynamic experiment, we set the values to be 4d and 3d, respectively, and d is the pixel size of the CCD.

According to the spectral range $\Delta\lambda_{window}$ used by the probe pulse, then the sequence

depth of the system can be calculated:

$$N = \frac{\Delta\lambda_{window}}{\delta\lambda} \tag{S13}$$

Time resolution of chirped pulse

Before calculating the time resolution, we need to make the following assumptions. The interaction between the ultrafast process and the observation pulse satisfies a linear relationship. In this paper, the change of side shock wave and surface reflectivity satisfies this relationship. The chirped pulse used in this paper is consistent with the previous work [4], but the spectral range (observation time window) is different according to the different ultrafast phenomena. Moreover, the spectral-temporal correspondence of the chirped pulse and the time range of the chirped pulse have been verified in advance by the optical Kerr effect [5]. According to the time resolution formula of chirped pulse single frame image [6–8]:

$$\tau = \sqrt[2]{\tau_p\tau_0 + (\Delta\omega\beta)^2} \tag{S14}$$

Where τ_p is the Fourier limit duration of the original femtosecond pulse, and τ_0 is the time range of the chirped pulse, $\Delta\omega$ is the frequency range of the single-frame observation pulse, which can be expressed as $\Delta\omega = -2\pi c\Delta\lambda/{\lambda_0}^2$, $\Delta\lambda$ is the spectral width of the single-frame observation pulse, λ_0 is the central wavelength of the laser, and β is group delay dispersion.

In the experiment of the side shock wave induced by femtosecond laser, the chirped pulse time range τ_0 is 256 ps, the spectral width of the single frame observation pulse $\Delta\lambda$ is 0.34 nm, and the center wavelength λ_0 is 800 nm. According to the above formula, the time resolution of the single frame image is calculated to be 5.27 ps.

In the experiment of femtosecond laser ablation of silicon surface reflectivity change, the chirped pulse time range τ_0 is 202 ps, the spectral width of the single frame observation pulse $\Delta\lambda$ is 0.3 nm, and the center wavelength λ_0 is 800 nm. According to the above formula, the time resolution of the single frame image is calculated to be 4.33 ps.

Note 6: Parameters of equipment

The light source used is a Ti: sapphire laser (Spectral Physics). The laser generates femtosecond pulses with a center wavelength of 800 nm, a pulse width of 35 fs, and a repetition rate of 1 kHz. At the same time, the laser can be set to single-shot mode, that is, only one pulse is emitted per trigger. The number of pairs of grating lines used in Fig. S3a is 1200 lp/mm (THORLABS, GR25-1208). The slit adjustable range is 0-13mm (Zolix, APAS80-1A). The TDG1 and TDG2 models are the same in Fig. S1a (Holoeye, DE-R 285). The overall size of the code is 1.2cm * 1.2cm, in which the micro-element size is 30 μm. The number of grating lines in Fig. S1a is 300 lp/mm (THORLABS, GR25-0310). CCD1 and CCD2 have the same pixel size (4096 * 3000, 3.45 μm * 3.45 μm, LUSTER LightTech, BFS-U3-122S6M).

In the experiment of femtosecond laser-induced side shock wave, a 10× objective (Nikon, 10×, NA = 0.25) focuses the pump pulse on the side of the material, and a 50× objective (DIMENSION, 50×, NA = 0.6) is used to amplify the ultrafast phenomenon. The size of the titanium alloy (Ti6A14V) used is 20 mm * 20 mm * 4 mm. The chemical composition (in mass percentage) of the titanium alloy is: 6.16 % aluminum, 3.95 % vanadium, 0.03 % iron, 0.04 % carbon, 0.06 % oxygen, 0.014 % nitrogen and the remaining titanium.

In the experiment of femtosecond laser ablation of silicon surface reflectivity changes, BBO crystal (CASTECH) is used to double the frequency of the pump pulse. The 20× objective lens (SOPTOP, 20×, NA = 0.45) is used to focus the pump pulse and image the reflected pulse. The polished silicon (100) used is 25.4 mm in diameter and 0.5 mm in thickness.

Additional supplementary pictures

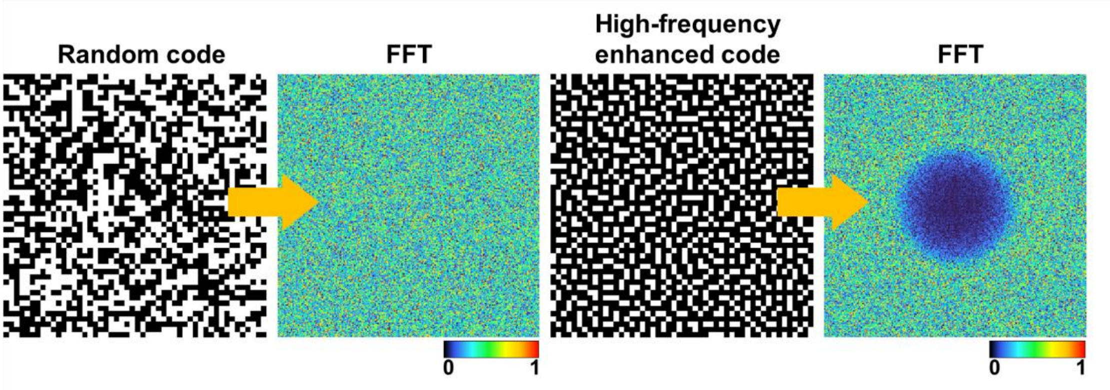

Fig. S1 Comparison of spatial distribution and frequency domain distribution of random code and high-frequency enhanced code. High-frequency code is more evenly distributed than random code, and there is no black and white pixel aggregation [4]. The high-frequency energy of the Fourier spectrum is stronger than the low-frequency energy, which can preserve the high-frequency information well when encoding objects.

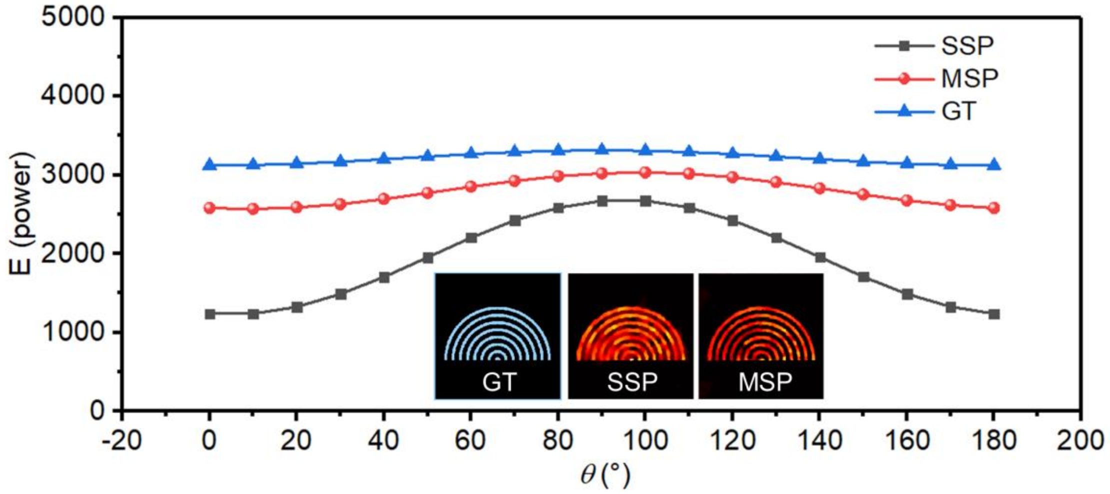

Fig. S2 The differential energy of the reconstruction results at different angles. When the pixel values of an image change relatively smoothly, the calculated total energy value will be lower. When there are sudden changes or obvious edges in the image, the calculated total energy will be larger. The high energy of MSP results represents high-quality images, which effectively reduces the blurring problem at different direction.

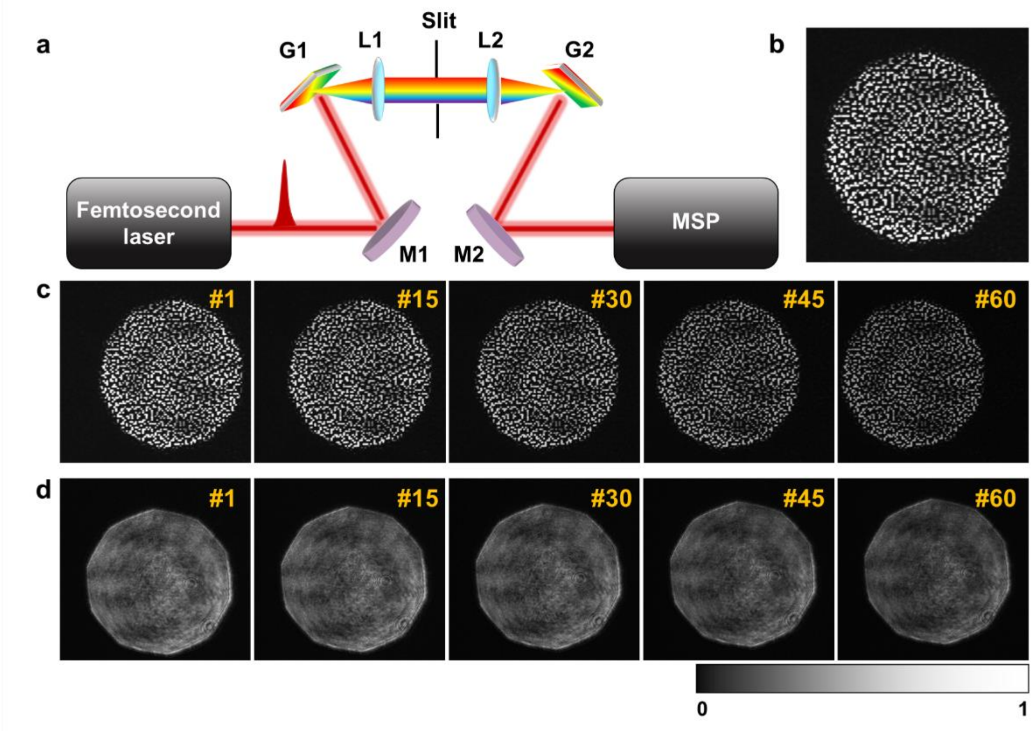

Fig. S3 a Spectral selection system. **b** Narrowing the slit width to obtain a clear coded image. **c** Calibration image of SSP. **d** Calibration image of MSP (only one of the projection dimensions).

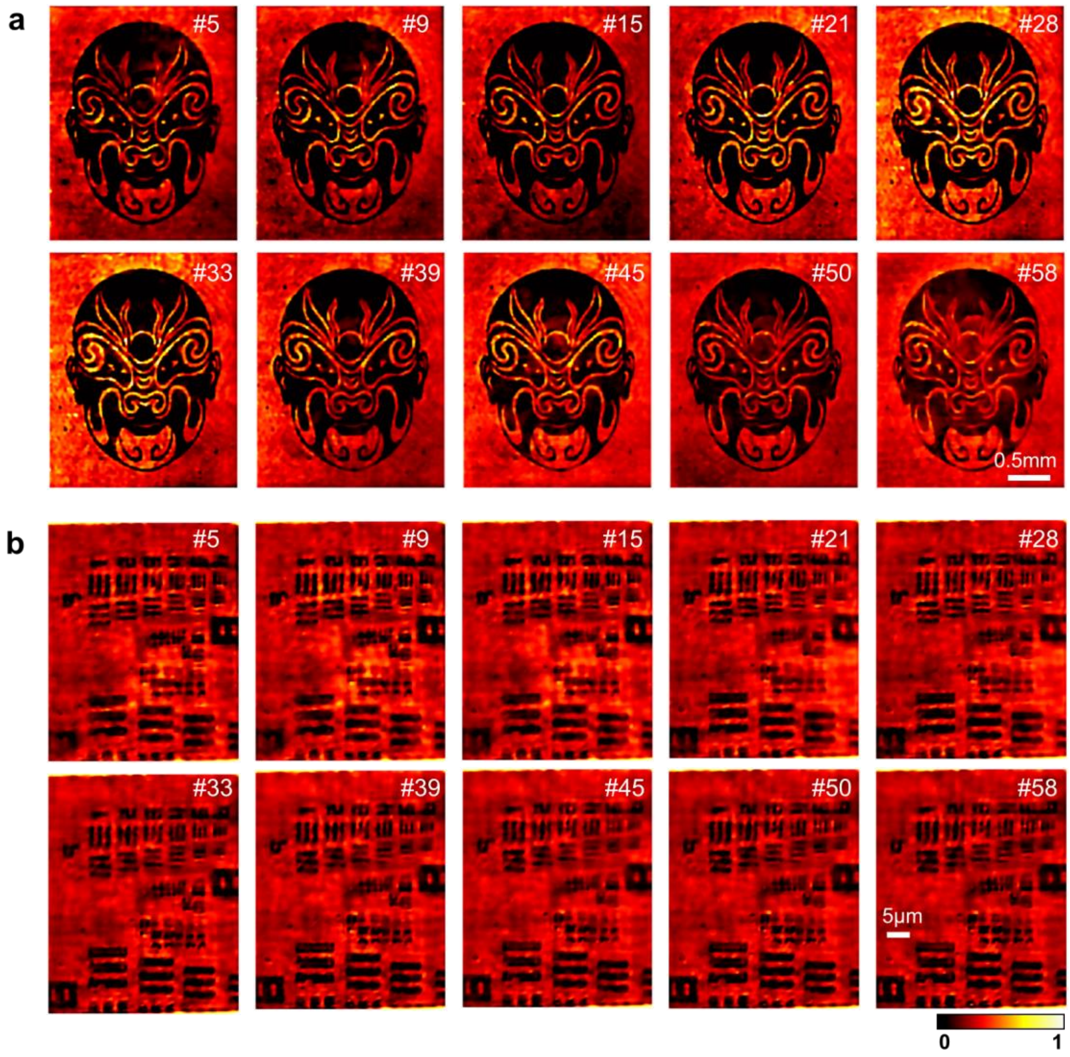

Fig. S4 a The reconstruction result of 'facial makeup' by MSP is displayed in one frame every six frames. **b** The reconstruction result of resolution plate by MSP is displayed in one frame every six frames.

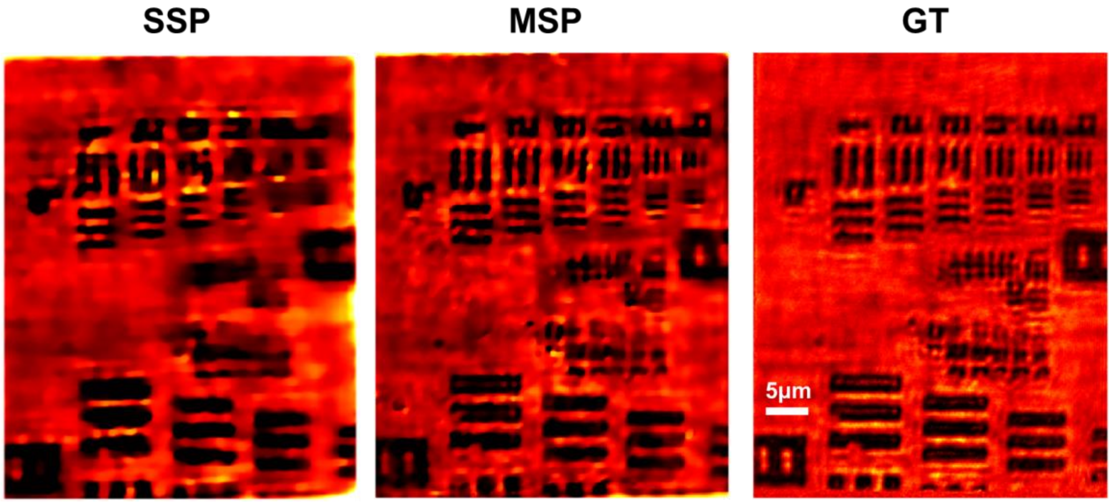

Fig. S5 The reconstruction results of SSP and MSP are compared with the Ground Truth (GT). From the GT, the imaging of the fifth element of the ninth group is indeed affected by the quality of resolution plate. The distinguishable resolution in the GT image is Group 10 Element 1(488 nm), which reaches the diffraction limit of the objective lens used (50×, NA=0.8).

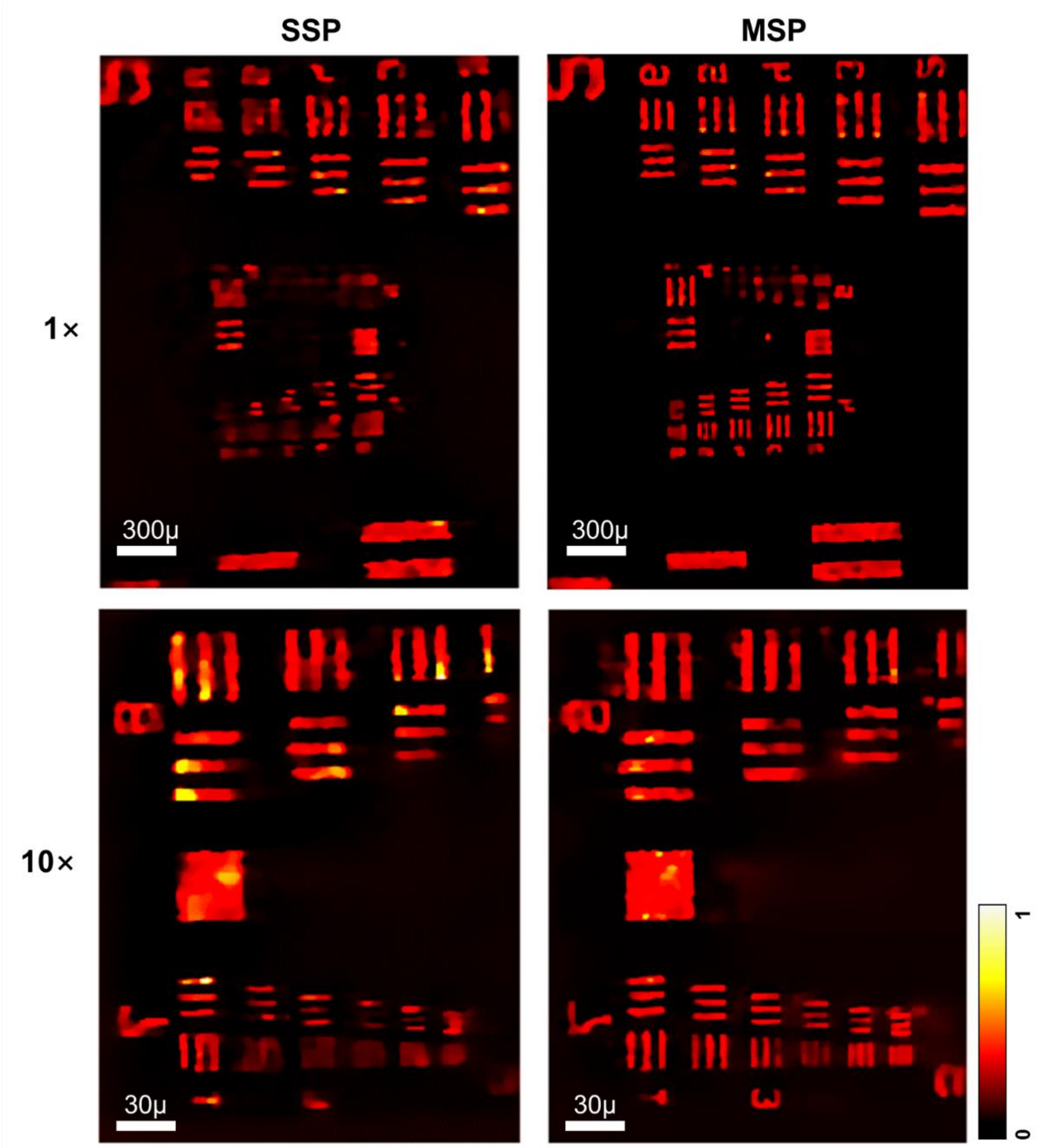

Fig. S6 The characterization of reconstructed spatial resolution of SSP and MSP at 1 and 10 magnifications.

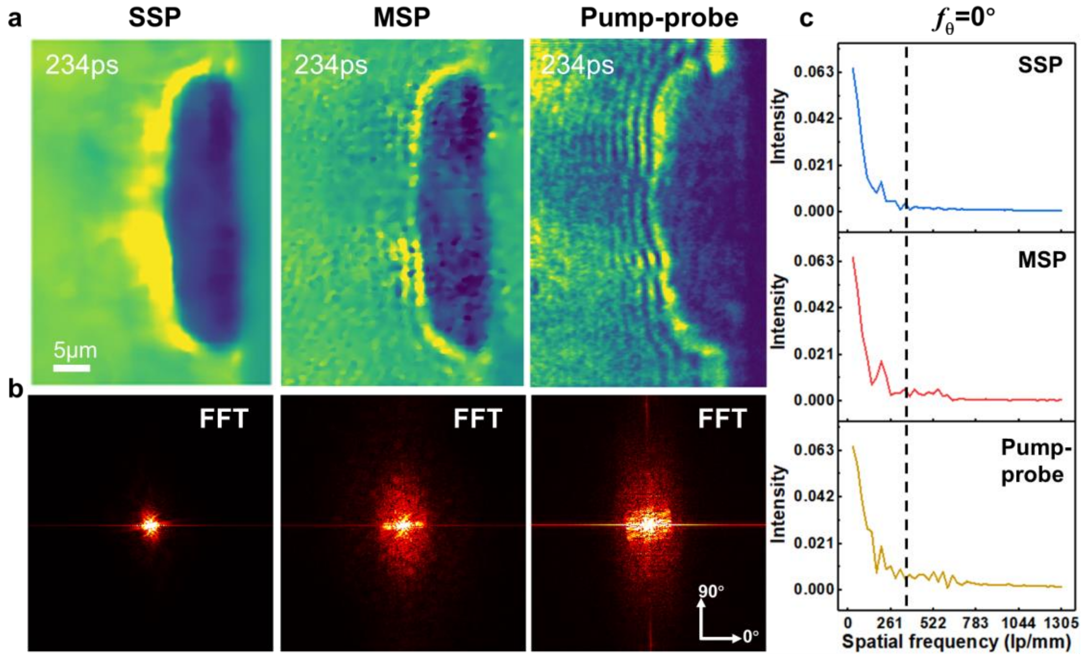

Fig. S7 a The reconstruction results of side shock wave expansion by SSP and MSP are compared with the results of pump-probe imaging. **b** Comparison of Fourier spectrum. **c** The frequency intensity curve extracted from the **b**, which shows that the extra frequency in the MSP is the real image detail.

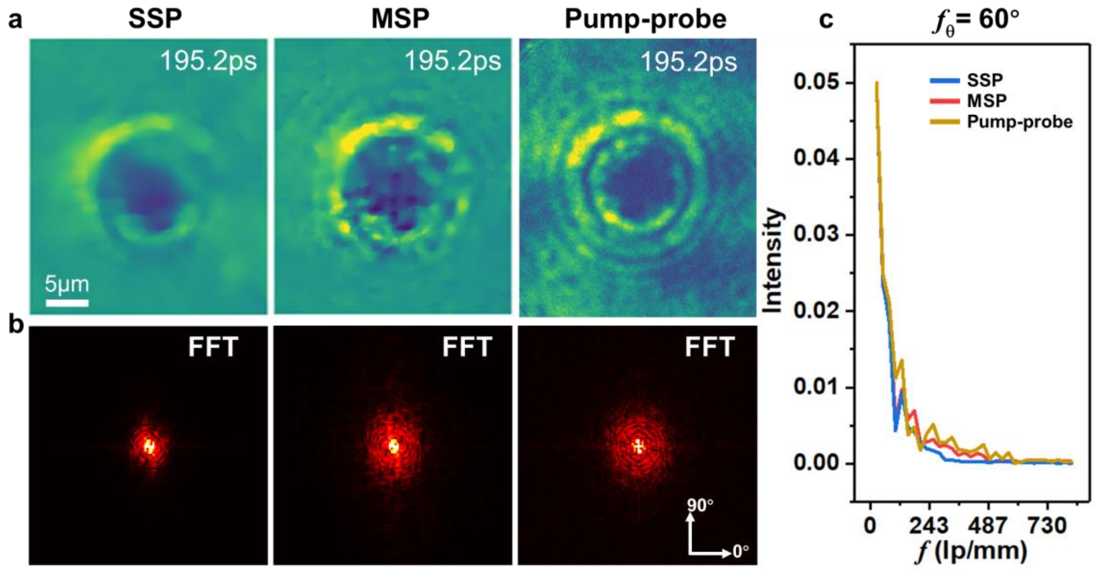

Fig. S8 a The reconstruction results of reflectivity change of femtosecond laser ablated silicon surface by SSP and MSP are compared with the results of pump-probe imaging. **b** Comparison of Fourier spectrum. **c** The frequency intensity curve extracted from the **b**.

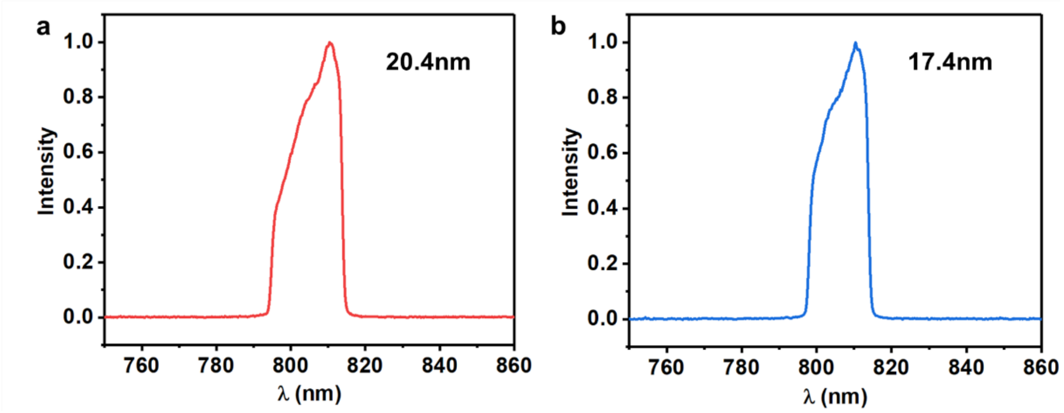

Fig. S9 a and **b** The spectral width of the chirped light used for femtosecond laser-induced side shock wave and femtosecond laser ablation of silicon surface reflectivity changes, respectively.

Supplementary Table

Method	Magnification	Spatial Resolution	Sequence Depth
**FISI**[9]	0.47×	9.84μm	6
**FINCOPA**[10]	1×	19.7μm	8
**DI-CUP**[11]	10×	11μm	16
**2DSMD-STAMP**[12]	20×	2.46μm	25
**SCARF**[13]	20×	2.2μm	23
**DMA-CSMUP**[14]	50×	0.83μm	42
**MSP (Ours)**	50×	0.62μm	60

Table 1. Comparison of MSP to representative single-shot ultrafast imaging techniques with high spatial resolution. FISI, frequency domain integration sequential imaging; FINCOPA, framing imaging based on cascaded non-collinear optical parametric amplification; DI-CUP, discrete illumination-based CUP; 2DSMD-STAMP, 2D spatial mapping device sequentially timed all-optical mapping photography; SCARF, swept coded aperture real-time femtophotography; DMA-CSMUP, demosaicing algorithm-based chirped spectral-mapping ultrafast photography.

Movie S1.

Continuous images of femtosecond laser-induced lateral shock waves reconstructed by SSP and MSP respectively. The number of frames collected is 60, and the time window is 256 ps.

Movie S2.

Continuous images of ultrafast evolution of silicon surface ablated by femtosecond laser reconstructed by SSP and MSP respectively. The number of frames collected is 60, and the time window is 202 ps.

References

1. Bioucas-Dias, J. M. & Figueiredo, M. A. T. A New TwIST: Two-Step Iterative Shrinkage/Thresholding Algorithms for Image Restoration. *IEEE Trans. on Image Process.* **16**, 2992–3004 (2007).
2. Gao, L., Liang, J., Li, C. & Wang, L. V. Single-shot compressed ultrafast photography at one hundred billion frames per second. *Nature* **516**, 74–77 (2014).
3. Sedov, L. I. SIMILARITY and DIMENSIONAL METHODS in MECHANICS.
4. Meng, Y. *et al.* High-frequency enhanced ultrafast compressed active photography. *OEA* **8**, 240180–240180 (2025).
5. Wang, L., Ho, P. P., Liu, C., Zhang, G. & Alfano, R. R. Ballistic 2-D Imaging Through Scattering Walls Using an Ultrafast Optical Kerr Gate. *Science* **253**, 769–771 (1991).
6. Sun, F. G., Jiang, Z. & Zhang, X.-C. Analysis of terahertz pulse measurement with a chirped probe beam. *Applied Physics Letters* **73**, 2233–2235 (1998).
7. Tang, H. *et al.* Temporal resolution of ultrafast compressive imaging using a single-chirped optical probe. *Opt. Lett.* **48**, 6080 (2023).
8. Lu, Y., Meng, Y., Yin, F., Yang, Q. & Chen, F. Single-Shot Spectral-Temporal Ultrafast Photography: Fundamentals, Methods, and Applications. *ACS Photonics* acsphotonics.5c00135 (2025) doi:10.1021/acsphotonics.5c00135.
9. Zhu, Q. *et al.* FISI: frequency domain integration sequential imaging at 1.26×10^{13} frames per second and 108 lines per millimeter. *Opt. Express* **30**, 27429 (2022).
10. Wang, C. *et al.* Single-shot 8-frame direct imaging with an effective frame rate up to 40 Tfps and a two-dimensional space-bandwidth-product beyond 65,500. *Opt. Express* **33**, 38088 (2025).

11. Yao, J. *et al.* Discrete Illumination-Based Compressed Ultrafast Photography for High-Fidelity Dynamic Imaging. *Advanced Science* **11**, 2403854 (2024).

12. Li, Z. *et al.* 2D spectrum slicing for sequentially timed all-optical mapping photography with 25 frames. *Applied Physics Letters* **123**, 141103 (2023).

13. Liu, J. *et al.* Swept coded aperture real-time femtophotography. *Nat Commun* **15**, 1589 (2024).

14. He, Y. *et al.* Single-shot high-fidelity large-sequence chirped spectral-mapping ultrafast photography. *Phys. Rev. Applied* **22**, 054058 (2024).
